# Caffeine-Induced Psychosis: A Case Report and Review of Literature

**DOI:** 10.7759/cureus.66306

**Published:** 2024-08-06

**Authors:** Dylan Mannix, Kate Mulholland, Fintan Byrne

**Affiliations:** 1 Psychiatry, University Hospital Galway, Galway, IRL; 2 Psychiatry, Mayo University Hospital, Castlebar, IRL

**Keywords:** withdrawal, over-the-counter drugs, anxiety, drug-induced psychosis, caffeine intoxication

## Abstract

A 51-year-old female, with no previous history of psychosis, presented to the Emergency Department with an acute psychotic episode in the context of excess caffeine consumption. Caffeine is an adenosine antagonist. An antagonist of adenosine can lead to the release of dopamine into the synaptic cleft, which can induce psychotic symptoms in vulnerable individuals. The patient had consumed caffeine in the form of up to eight energy drinks daily. She experienced persecutory delusions alongside auditory and visual hallucinations. She did not have a history of psychotic disorder but did have a history of generalized anxiety disorder. Upon cessation of caffeine, her symptoms resolved within five days. She remained caffeine-free and symptom-free 18 months later when reviewed in the community. This case highlights the potential psychiatric consequences of excessive caffeine consumption and identifies the need to screen for excessive consumption of caffeine in individuals presenting with new psychotic symptoms or worsening of pre-existing psychotic symptoms.

## Introduction

Caffeine is the most commonly used psychoactive substance worldwide, with about 85% of the US population consuming at least one caffeinated beverage per day [1]. It is frequently consumed in coffee, where standard doses are between 80 and 175 mg per cup [2], and it is also available in soft drinks, energy drinks, tea, chocolate, and over-the-counter medications.

Caffeine exerts its psychoactive effects primarily by antagonizing adenosine receptors, which leads to increased release of neurotransmitters such as dopamine and glutamate. This mechanism can result in heightened alertness and improved cognitive performance at moderate doses but may contribute to anxiety, insomnia, and even psychosis at higher doses [3].

The subject of our case study had been consuming “Red Berry Boost,” an energy drink containing 30 mg/100 mL, available in 250 mL and 500 mL containers. Lethal doses of caffeine are typically between 150 and 200 mg per kg of body weight [4]. Previous reports have indicated that caffeine may exacerbate psychosis at doses of 10 mg per kg in individuals with a history of schizophrenia [5]. A recent case described the onset of psychotic symptoms following coffee consumption in a 12-year-old boy; however, this child also manifested significant physical symptoms [6]. Another case described in the literature described the onset of mania in a man who had been consuming excessive amounts of energy drinks; however, this man also had a history of illicit drug use, which is absent in this case [7]. Caffeine-induced psychosis is rare in people who do not have pre-existing psychotic illnesses.

According to the ICD-11 (eleventh revision of the International Classification of Diseases), caffeine intoxication usually occurs at doses greater than 1 g per day [8]. However, the woman in this case was consuming approximately 640 mg daily, suggesting that pre-existing vulnerabilities may lower the threshold for caffeine-induced psychosis. The patient in this case manifested some features of ICD-11 caffeine intoxication such as perceptual disturbances, changes in affect, and behavioral symptoms. There is no specific ICD-11 code for caffeine-induced psychosis.

## Case presentation

We present the case of a 51-year-old, separated, Caucasian female who presented to the Emergency Department following the acute onset of paranoid delusions and auditory hallucinations. She had a psychiatric history of generalized anxiety disorder for which she was at the time of initial presentation not receiving any psychological or pharmacological intervention. She reported the onset of symptoms five days previously. The symptoms she reported were auditory hallucinations of indistinct voices and fears that others might wish to harm her. She disclosed consuming two cans of energy drink per day at this time, which equated to a total of 80 mg of caffeine. She was commenced on sertraline to be taken in the morning and quetiapine to be taken at night. These medications were increased to 100 mg and 150 mg, respectively. Within one week her symptoms had resolved. During this time she had ceased her intake of energy drinks. Both pharmacological interventions were continued on resolution of her symptoms.

Five months later, the patient re-presented to the psychiatry service with a relapse of psychotic symptoms and prominent anxiety symptoms, worsening over the course of seven days. She reported paranoid persecutory delusions, believing her neighbors might enter her home to kill her. She experienced auditory hallucinations in the second person, in the form of unrecognizable male voices. She said these voices had a threatening nature and made derogatory comments toward her. She also reported visual hallucinations of shadows in her peripheral vision. She did not experience perceptual abnormalities in other modalities. The patient was distressed by the perceptual abnormalities. She reported a deterioration in somatic anxiety symptoms, with increased shortness of breath, palpitations, and muscle tension. There had been a progressive change in behavior in the week prior to the presentation.

Collateral information obtained from the patient’s daughter endorsed social withdrawal and hypervigilance, resulting in the patient leaving her own home to live with her daughter. The patient also reported poor appetite, concentration, and a dysregulated sleeping pattern secondary to auditory hallucinations. She denied a history of alcohol use or illicit substance use; however, she disclosed consuming energy drinks daily. Consumption of energy drinks had gradually increased from two daily at the initial presentation to eight energy drinks daily, which equated to a total of 600 mg of caffeine per day.

The patient accepted an admission to the inpatient psychiatric unit. On physical examination, she had a resting pulse rate of 112 bpm, respiratory rate of 25 rpm, and blood pressure of 145/90 mmHg. The patient had no medical history of note. There was a family history of depression and completed suicide (first cousin) and anxiety disorder (paternal aunt). There was no known family history of psychotic illness.

Investigations

A full range of blood tests including full blood count, urea and electrolytes, liver function tests, and c-reactive protein were carried out on admission. This revealed elevated creatine kinase at 220 IU/mmol likely linked to the patient’s consumption of energy drinks. Other blood analyses were unremarkable, and a urine toxicology screen was negative.

Treatment

Following admission to the inpatient psychiatric unit, the patient continued quetiapine 150 mg and sertraline 100 mg, and the consumption of energy drinks was stopped. The patient was not prescribed any further medication in addition to the aforementioned. The patient was allowed to consume three cups of tea per day to mitigate some of her caffeine withdrawal symptoms. Initially, the patient presented with psychomotor agitation, increased anxiety, and hypervigilance on the ward. These symptoms gradually settled over the course of five days. She was apprehensive regarding discharge and fearful of a reoccurrence of symptoms. Ten days after admission, there was no evidence of psychotic symptoms, and the patient was discharged home with planned community mental health team follow-up.

Outcome and follow-up

The psychotic symptoms had resolved within five days of admission following the cessation of consumption of energy drink consumption, with no change to psychotropic medications. During the subsequent outpatient appointments over the course of 18 months, the patient abstained from energy drink consumption and has had no relapse in psychotic symptoms. The patient continued to consume up to three cups of tea per day. She does not drink coffee. The patient continues to attend the outpatient clinic for the treatment of generalized anxiety disorder which is well-controlled. She continues to be prescribed sertraline 100 mg daily and quetiapine 150 mg at night.

## Discussion

Caffeine-induced psychosis has been described in the literature on multiple previous occasions. The majority of these cases occur in people who had preexisting psychotic illnesses, with five cases being described in people who did not have preexisting psychotic illnesses [9]. This is the second case of caffeine-induced psychosis in a woman who did not have a chronic psychotic illness [10], although the woman in our case did describe psychotic phenomena on a previous emergency department presentation in the context of caffeine consumption.

Caffeine acts as a competitive antagonist at all adenosine receptors. The relevance of caffeine in this case is in relation to its activity at the adenosine A1 and A2A receptor. Adenosine receptors are G-protein-coupled receptors expressed in the central and peripheral nervous systems and in the cardiovascular system. Adenosine metabolism is closely linked to dopamine metabolism, and it is possible for adenosine and dopamine receptors to form heteromers [11]. Agonism of the adenosine receptor reduces dopamine release. Antagonism of the A2A receptor can induce dopamine activity at D2 receptors. Antagonism of the A1 receptor interacts with D1 receptors and regulates the release of additional neurotransmitters such as dopamine, glutamate, and acetylcholine [12]. This effect can promote wakefulness and induce positive emotions but also can induce psychotic and manic symptoms in vulnerable individuals [5-7]. There is known genetic variability in the adenosine receptor. Common variants of the gene encoding the A2A receptor can disrupt sleep or cause anxiety after the ingestion of caffeine [13].

In regard to the neuropharmacological effects of caffeine, one study showed that caffeine-induced dopamine and glutamate release in the shell of the nucleus accumbens [3]. This effect was replicated by administration of a selective A1 adenosine antagonist but not an A2A adenosine receptor antagonist. Other psychostimulant drugs, such as amphetamines and cocaine, have also been shown to increase the extracellular concentration of dopamine in the nucleus accumbens [9]. One study demonstrated that over the course of long-term exposure, the dopamine release effects in the nucleus accumbens are attenuated via an A1 receptor-mediated mechanism, representing the development of tolerance [14]. Another study found that caffeine actually modulates the availability and affinity of postsynaptic dopamine receptors and that this increased availability is responsible for the increased dopaminergic activity. Specifically, caffeine increases D2 and D3 receptor availability and affinity in the striatum [15]. This is similar to a recent article published in the Schizophrenia Bulletin, which describes increased post-synaptic receptor availability and affinity in people who have been exposed to long-term antipsychotics and then have same withdrawn, causing a relapse of psychosis via drug-induced dopaminergic hypersensitivity [16].

Caffeine is both water and fat soluble and easily crosses the blood-brain barrier. Caffeine can cross the placenta and is also excreted in breast milk. When ingested orally, caffeine is absorbed fully within 1 hour and then rapidly diffuses into other tissues. This rate of absorption does not vary with demographic factors such as age, race, and sex. Peak serum concentrations of caffeine are reached 2 hours following ingestion. Caffeine has a long half-life of 3-7 hours. Caffeine’s metabolite, paraxanthine, has also been shown to increase extracellular levels of dopamine via inhibition of phosphodiesterase [13].

Caffeine is metabolized by the CYP1A2 enzyme of the cytochrome P450 family. There are several factors which influence caffeine metabolism, including genetic variability in the *CYP1A2* gene. There are at last 150 known single-nucleotide polymorphisms that can accelerate caffeine clearance. The half-life of caffeine may be increased in liver diseases which decrease P450 activity. The susceptibility to caffeine’s harmful effects may be greater in those who have not developed pharmacological tolerance, such as children and adolescents [3].

One study found that caffeine consumption reduced cognitive decline in women but not in men [17]. Large cohort studies of both men and women have found an inverse relationship between caffeine consumption and the risk of Parkinson’s disease and Alzheimer’s disease [18].

The DSM-5 (Diagnostic and Statistical Manual of Mental Disorders, Fifth Edition) includes a section on caffeine use disorder, which is similar to the caffeine dependence syndrome in the ICD-10 (tenth revision of the International Classification of Diseases) [19,20]. The DSM-5 criteria overlap significantly with other substance use disorders. Criteria are persistent desire or unsuccessful attempts to cut back, continued use despite harmful effects, and withdrawal symptoms. All three of these criteria must be met for the diagnosis of caffeine dependence with additional criteria, i.e. tolerance, craving, and impact on socio-occupational functioning being used to categorize severity. The ICD-11 section on disorders due to caffeine use includes episode of harmful caffeine use, harmful pattern of caffeine use, caffeine intoxication, caffeine withdrawal, and residual categories. ICD-11 does not have a specific entry available for caffeine-induced psychosis. "Psychotic disorder induced by other specified psychoactive substance 6C4E.6" and “Caffeine intoxication 6C48.2” are the ICD-11 diagnoses relevant to this case. The essential criteria for caffeine intoxication include “transient and clinically significant disturbances in consciousness, cognition, perception, affect, behavior, or coordination that develop during or shortly after the consumption or administration of caffeine." Additional essential criteria for caffeine intoxication include that the symptoms must abate on clearance of caffeine from the patient’s metabolism, and that the symptoms must be in keeping with known physiological effects of caffeine [8].

## Conclusions

In this article, we have described a case of induction of psychotic symptoms in a woman with preexisting vulnerabilities, i.e., a history of persecutory ideas, perceptual disturbances, and a diagnosis of generalized anxiety disorder. This case highlights that over-the-counter preparations, which may not be considered potently psychoactive by the general population, can have relevance in psychiatric practice because of their ability to induce psychopathology characteristic of severe conditions such as schizophrenia. The authors argue that it is worthwhile to ask simple screening questions to assess the quantity of caffeine consumed by people presenting with new psychotic symptoms, or deterioration of psychotic symptoms in individuals with chronic psychotic illnesses. Further research is required to determine if it is worthwhile to advise people with chronic psychotic illnesses to minimize their caffeine intake to reduce symptom burden.
